# Supplementary material for: Secretory leukocyte protease inhibitor (SLPI) promotes cholangiocarcinoma progression via inflammation-associated and vasculogenic mechanisms
Source: PLoS One. 2026 Jan 20;21(1):e0340763. doi: 10.1371/journal.pone.0340763 (PMC12818638; doi:10.1371/journal.pone.0340763)
Supplement: Attachment — Submitted filename: Response to Reviewers.docx [file pone.0340763.s007.docx]

PONE-D-25-54451

**Title:** *Secretory leukocyte protease inhibitor (SLPI) promotes cholangiocarcinoma progression via inflammation-associated and vasculogenic mechanisms*

**Point by point respond to Reviewer’s comments**

**Reviewer 1**

Chueajedton et al described Secretory leukocyte protease inhibitor (SLPI) promotes cholangiocarcinoma progression via inflammation-associated and vasculogenic mechanisms. The general concept is interesting and just some minor changes should be made to improve the manuscript.

|  | **Comments** | **Responses** |
| --- | --- | --- |
| 1 | Could the authors elaborate on how SLPI mechanistically links inflammation to vasculogenic mimicry in cholangiocarcinoma? The transition between these two roles seems conceptually broad. | We thank the reviewer for this insightful comment. We agree that the mechanistic link between inflammation-associated SLPI induction and vasculogenic mimicry (VM) warrants clearer explanation.  In the revised Discussion, we have expanded the mechanistic framework to clarify how inflammation-induced SLPI may bridge these processes. Briefly, chronic inflammatory signaling, particularly IL-6–mediated NF-κB activation, induces SLPI expression in cholangiocytes and CCA cells. Beyond its classical anti-protease role, SLPI has been reported to function as a signaling modulator that promotes tumor cell plasticity and extracellular matrix remodeling.  Our data show that SLPI overexpression enhances MMP-9 activity, increases VEGFA and VE-cadherin expression, and suppresses N-cadherin, a molecular signature consistent with VM-competent tumor cells rather than endothelial-driven angiogenesis. These changes suggest that SLPI facilitates a phenotypic shift in CCA cells toward an endothelial-like state capable of forming perfusable, vascular-like channels independent of endothelial cells.  Moreover, previous studies have demonstrated that SLPI can activate oncogenic signaling pathways such as STAT3, RB/FOXM1, and PI3K/AKT, which are implicated in tumor cell survival, invasion, and vasculogenic mimicry formation. We therefore propose that SLPI acts as an inflammation-responsive mediator that integrates IL-6/NF-κB signaling with downstream transcriptional and proteolytic programs, enabling CCA cells to adopt VM-associated phenotypes.  We have revised the Discussion to explicitly describe this mechanistic continuum—from inflammation-induced SLPI upregulation to VM formation—and to distinguish VM from classical angiogenesis in the context of CCA progression (Discussion, pages 19, line 427-441). |
| 2 | Were any control experiments performed to rule out off-target effects of SLPI overexpression, especially in the context of MMP activation and VM formation? | We thank the reviewer for this important question regarding the specificity of SLPI overexpression–mediated effects.  To minimize potential off-target effects, several experimental design features were incorporated in this study. First, SLPI overexpression was achieved using stable transfection followed by antibiotic selection, and two independent SLPI-overexpressing clones [SLPI(1) and SLPI(2)] were generated and analyzed. Both clones consistently exhibited similar trends in tumorigenic, metastatic, MMP activation, and vasculogenic mimicry (VM) phenotypes, supporting the reproducibility and specificity of SLPI-associated effects rather than clone-specific artifacts.  Second, phenotypic and molecular changes observed in SLPI-overexpressing cholangiocytes were benchmarked against endogenous SLPI-high cholangiocarcinoma cell lines (KKU-213A and KKU-213AL5). Notably, increased MMP-9 activity and enhanced VM formation in SLPI-overexpressing cells mirrored those observed in metastatic CCA cells with naturally high SLPI expression, further supporting a biologically relevant SLPI-dependent effect.  Third, we demonstrated that inflammatory stimulation with IL-6, an upstream physiological inducer of SLPI, similarly increased SLPI expression and promoted tumorigenic properties, providing an independent, non-genetic line of evidence linking SLPI induction to malignant phenotypes.  While loss-of-function approaches (e.g., siRNA-mediated knockdown or neutralization of SLPI) would provide additional confirmation of causality, these experiments were beyond the scope of the present study and are acknowledged as a limitation. We have clarified this point in the Discussion and highlighted SLPI loss-of-function studies as an important direction for future research. (Discussion, pages 21, line 470-477). |
| 3 | The manuscript mentions IL-6 stimulation increasing SLPI expression. Was the IL-6 concentration physiologically relevant, and were time-course experiments conducted? | We thank the reviewer for this important comment regarding the physiological relevance of IL-6 stimulation.  The IL-6 concentrations used in this study (1–10 ng/mL) are within the range commonly employed to model chronic inflammatory signaling in vitro and have been widely reported to activate NF-κB signaling and downstream inflammatory responses in cholangiocytes and cholangiocarcinoma cells. Importantly, elevated IL-6 levels have been detected in the serum and tumor microenvironment of patients with cholangiocarcinoma, particularly in inflammation-associated disease contexts such as Opisthorchis viverrini infection.  To address temporal regulation, we performed time-course experiments at 12 and 24 hours. These time points were selected to capture both early and sustained inflammatory responses. Our results demonstrate that IL-6 induces NF-κB activation followed by increased SLPI expression in a dose- and time-dependent manner, supporting a biologically relevant and sustained inflammatory induction rather than an acute, transient effect.  Together, these findings support the use of IL-6 stimulation as a physiologically relevant in vitro model of inflammation-associated cholangiocarcinogenesis. We have clarified the rationale for IL-6 dose selection and time-course design in the Discussion sections. (Discussion, pages 18, line 390-396). |
| 4 | Given the use of both hamster and mouse models, how do the authors reconcile interspecies differences in SLPI expression and tumor microenvironment? | We thank the reviewer for raising this important point regarding interspecies differences.  In this study, hamster and mouse models were used to address complementary biological questions rather than to directly compare species-specific tumor microenvironments. The hamster model was employed to recapitulate inflammation-associated cholangiocarcinogenesis driven by *Opisthorchis viverrini* infection, which closely mirrors the etiological and pathological features of human cholangiocarcinoma endemic to Southeast Asia. In contrast, the mouse xenograft model was used to evaluate the tumorigenic consequences of SLPI overexpression in vivo under controlled experimental conditions.  Importantly, our conclusions regarding SLPI are primarily supported by consistent findings across multiple experimental platforms, including human transcriptomic datasets, human cholangiocyte and cholangiocarcinoma cell lines, and both animal models. In the hamster model, SLPI expression progressively increased during inflammation-driven CCA development, while in the mouse xenograft model, enforced SLPI expression enhanced tumor growth. These concordant trends suggest that the tumor-promoting role of SLPI is conserved across species, despite inherent differences in immune context and stromal composition.  We acknowledge that species-specific differences in immune responses and tumor microenvironment may influence the magnitude and dynamics of SLPI signaling. However, the integration of inflammation-driven (hamster), cell-autonomous tumorigenicity (mouse xenograft), and human clinical data strengthens the translational relevance of our findings. We have clarified this rationale and acknowledged interspecies differences as a limitation in the revised Discussion. (Discussion, pages 21 line 477-485). |
| 5 | The authors state that SLPI-overexpressing cells did not affect angiogenesis but promoted VM. Was endothelial tube formation quantified, and how was VM distinguished from true angiogenesis? | We thank the reviewer for this important question regarding the distinction between angiogenesis and vasculogenic mimicry (VM).  In this study, angiogenesis and VM were assessed using distinct experimental systems designed to specifically interrogate endothelial-dependent versus tumor cell–intrinsic vascular-like behaviors. Angiogenesis was evaluated using EA.hy926 endothelial cells cultured on Matrigel and exposed to conditioned media from Mock or SLPI-overexpressing cholangiocytes. Endothelial tube formation was quantified by counting tubular structures formed by EA.hy926 cells under identical conditions. No significant differences were observed in endothelial proliferation, migration, or tube formation between conditioned media from Mock and SLPI-overexpressing cells, indicating that SLPI does not promote classical endothelial-driven angiogenesis.  In contrast, vasculogenic mimicry was assessed by seeding tumor cells directly onto Matrigel under hypoxic conditions, where endothelial cells were absent. VM structures were defined as interconnected, tubular-like networks formed by tumor cells themselves. These structures were quantified by manual counting of tubular formations per field, following established VM assay protocols. SLPI-overexpressing cells exhibited a marked increase in VM structures compared with Mock controls.  Importantly, the VM phenotype was further supported at the molecular level by increased expression of VE-cadherin and VEGFA and reduced N-cadherin expression, a molecular signature characteristic of VM-forming tumor cells rather than endothelial angiogenesis. We have clarified the quantification criteria and explicitly distinguished VM from angiogenesis in the Methods and Figure 6 legend. |
| 6 | The bioinformatic correlation between SLPI and poor survival is compelling. Were multivariate analyses performed to control for confounding clinical variables? | We thank the reviewer for this important comment regarding survival analysis.  In the present study, the survival analysis was performed using publicly available transcriptomic datasets accessed through the GEPIA2 platform, which provides univariate Kaplan–Meier survival estimates based on gene expression levels. Unfortunately, detailed clinicopathological variables (e.g., tumor stage, grade, treatment modality, or comorbidities) required for multivariate Cox regression analysis were not available within this dataset. Therefore, multivariate survival analysis could not be reliably performed in the current framework.  Nevertheless, the observed association between high SLPI expression and poor patient survival was consistent with its progressive upregulation during cholangiocarcinogenesis in the hamster model and its tumor-promoting functions demonstrated in vitro and in vivo. Together, these complementary lines of evidence support a biologically meaningful role of SLPI in CCA progression.  We acknowledge the lack of multivariate survival analysis as a limitation of this study and have clarified this point in the Discussion. Future studies using well-annotated clinical cohorts with comprehensive clinicopathological data will be required to determine whether SLPI serves as an independent prognostic factor in cholangiocarcinoma. (Discussion, pages 22, line 486-490). |
| 7 | Do the authors envision SLPI as a diagnostic biomarker, therapeutic target, or both? Has its expression been validated in patient biopsies beyond bioinformatics? | We thank the reviewer for this important question regarding the translational relevance of SLPI.  Based on the current findings, we envision SLPI primarily as a promising diagnostic and prognostic biomarker, with potential therapeutic relevance that warrants further investigation. In this study, SLPI expression was consistently elevated during cholangiocarcinoma progression in human transcriptomic datasets, in an inflammation-driven hamster model, and in human cholangiocarcinoma cell lines. These convergent data support the clinical relevance of SLPI as a biomarker associated with disease progression.  Importantly, SLPI expression has also been validated at the tissue and serum levels in cholangiocarcinoma patients in our recent clinical and functional study, which demonstrated significantly higher SLPI expression in CCA tissues and sera compared with non-tumor controls and hepatocellular carcinoma, as well as functional evidence supporting its role in promoting CCA cell proliferation. These findings strengthen the biomarker potential of SLPI beyond bioinformatic analyses [22].  With respect to therapeutic targeting, our functional data demonstrate that SLPI overexpression promotes tumorigenic, metastatic, and vasculogenic mimicry–associated phenotypes, suggesting that SLPI may represent a candidate therapeutic target. However, direct therapeutic intervention strategies, such as SLPI inhibition or neutralization, were not evaluated in this study. Therefore, we consider the therapeutic potential of SLPI as a future direction that will require dedicated preclinical studies to assess efficacy and safety.  We have clarified this distinction between biomarker utility and therapeutic potential in the revised Discussion. (Discussion, pages 20, line 442-449) |
| 8 | Language and Structure  While the manuscript is scientifically sound, some sections (e.g., Methods and Results) could benefit from clearer transitions and more concise phrasing. Would the editor consider recommending language polishing? | We thank the reviewer for this constructive suggestion. In response, we have carefully revised the manuscript to improve clarity, coherence, and conciseness, particularly in the Methods and Results sections. Transitions between experimental steps and result subsections have been strengthened, redundant phrasing has been reduced, and terminology has been standardized throughout the manuscript to enhance readability.  These revisions were performed by the authors with careful attention to scientific accuracy and consistency, and no changes were made to the experimental design or interpretation of the data. We believe that these improvements have significantly enhanced the overall structure and readability of the manuscript. |

**Reviewer 2**

This manuscript investigates the role of SLPI in cholangiocarcinoma progression. The authors effectively combine clinical data (human tissues and bioinformatics), an in vivo hamster model, and a range of in vitro functional assays to build a strong case for SLPI as a promoter of tumorigenesis, metastasis, and vasculogenic mimicry The study addresses an important knowledge gap in understanding inflammation-associated cholangiocarcinoma progression. However, several methodological details and statistical analyses need clarification, and figure presentation must be substantially improved.

|  | **Comments** | **Responses** |
| --- | --- | --- |
| 1 | The authors should explain the tumor weight/volume discrepancy in Fig 4c-e. | We thank the reviewer for highlighting this important point. The observed discrepancy between tumor weight and tumor volume in the xenograft model likely reflects biological and methodological differences between these measurements rather than inconsistency in the data.  Tumor volume was estimated longitudinally using caliper measurements and the standard formula (V = ½ × L × W²), which assumes a relatively regular tumor geometry and does not account for intratumoral heterogeneity. In contrast, tumor weight was measured directly at the experimental endpoint and more accurately reflects total tumor mass, including contributions from necrotic areas, stromal components, edema, and extracellular matrix deposition.  In SLPI-overexpressing tumors, enhanced tumorigenic activity may promote increased cellular density and stromal remodeling without a proportional increase in external tumor dimensions, resulting in significantly higher tumor weight despite comparable measured volumes. Similar discrepancies between tumor volume and weight have been reported in xenograft studies where tumor composition differs between experimental groups.  We have clarified this point in the Results and discussed it as a limitation in the Discussion, noting that future studies with longer follow-up, imaging-based volume assessment, or histological quantification of tumor composition would further resolve this discrepancy. (Results, pages 14, line 314-317; Discussion, pages 21, line 464-467). |
| 2 | In vasculogenic mimicry assay (Fig 6d), the result is dramatic (279 vs. 46 structures) but the methodology is vague. How were these "tubular structures" defined and quantified? A clear description of the quantification method (e.g., number of tubes, junctions, total tube length using software like ImageJ) is necessary for reproducibility. | We thank the reviewer for this important comment regarding the quantification of tube formation and vasculogenic mimicry (VM) assays. Upon careful verification of our experimental records, we would like to clarify that both endothelial tube formation and VM formation were quantified using ImageJ software.  In the revised manuscript, we have corrected the Methods section to accurately describe the quantification approaches. For the tube formation assay, images of endothelial tubular-like structures were analyzed using ImageJ software to quantify the number of complete tubular structures per field under identical imaging conditions.  Similarly, VM formation was quantified using ImageJ software by analyzing tumor cell–derived tubular-like networks formed in the absence of endothelial cells. VM structures were defined based on their interconnected, tubular morphology, and quantification was performed in at least five randomly selected fields per well from three independent experiments.  These corrections improve methodological accuracy and transparency without affecting the results or conclusions of the study. (Methods, pages 8, line 171-184) |
| 3 | For statistical analysis, the description is adequate, but it should be explicitly stated whether the data met the assumptions of the tests used (e.g., normality for t-test/ANOVA). The use of "n" should be clarified for each experiment (e.g., n = 3 independent experiments, n=9 mice per group). Are “n” "three independent experiments" vs. "3 technical replicates from one experiment"? Post-hoc test specifications for ANOVA are missing for few results. | We thank the reviewer for this valuable comment. We agree that clearer reporting of statistical assumptions, the definition of “n”, and post-hoc testing improves transparency and reproducibility.  In the revised manuscript, we have updated the Statistical analysis section to explicitly state how assumptions were evaluated. Normality was assessed prior to parametric testing, and appropriate alternative analyses were considered when assumptions were not met. We also clarified the meaning of “n” throughout the manuscript, specifying whether it refers to independent biological experiments (for in vitro assays) or number of animals (for in vivo studies), and we differentiated biological replicates from technical replicates where applicable.  In addition, we specified the post-hoc test used following one-way ANOVA (e.g., Tukey’s multiple comparisons test) in the Statistical analysis section and in relevant figure legends to ensure consistency across reported results. (Methods, pages 10, line 212-224; Figure legends 1,3,5) |
| 4 | Figures are of very low resolution. Figures must be uploaded in high resolution. | We thank the reviewer for this comment. We have now replaced all figures with high-resolution versions in accordance with PLOS ONE guidelines. All figures have been uploaded as high-quality TIFF files at a minimum resolution of 300 dpi, with appropriate dimensions to ensure clarity of text, symbols, and graphical elements. We confirm that the revised figures meet the journal’s technical requirements and substantially improve visual quality and readability. |

**Reviewer 3**

Kangsadan et al in the manuscript titled “Secretory leukocyte protease inhibitor (SLPI) promotes cholangiocarcinoma progression via inflammation-associated and vasculogenic mechanisms” have attempted to understand the role of SLPI in cholangiocarcinoma.

Comments:

|  | **Comments** | **Responses** |
| --- | --- | --- |
| 1 | The role of SLPI in cancer has been extensively studied which is also mentioned by the authors, so the work adds to the knowledge database showing its relevance in cholangiocarcinoma as well. Although a variety of methods have been adopted, the manuscript lacks overall clarity.  The experimental workflow or the methodology section needs to be reorganized, adding more details on each assay, the cell line lines used etc. to better understand the sequence of experimental design. | We thank the reviewer for this constructive comment and for recognizing the relevance of our work in extending the role of SLPI to cholangiocarcinoma.  In response to this suggestion, we have revised the manuscript to improve overall clarity and organization, particularly within the Methods section. The experimental workflow has been reorganized to follow a more logical sequence, beginning with bioinformatic and clinical analyses, followed by inflammation modeling, functional in vitro assays, and in vivo studies. Additional methodological details have been provided for each assay, including cell line identities, experimental grouping, treatment conditions, and quantification approaches, to facilitate reproducibility and improve readability.  Furthermore, we have improved transitions between the Methods and Results sections to better reflect the progression of the experimental design. These revisions were aimed at enhancing clarity without altering the scientific content or conclusions of the study. |
| 2 | The study also lacks loss of function experiments to validate the obtained results but this has been mentioned by the authors as a limitation of the study. | We thank the reviewer for this comment and for acknowledging that the lack of loss-of-function experiments has been recognized as a limitation of the present study.  We agree that complementary loss-of-function approaches, such as SLPI knockdown or neutralization, would provide additional mechanistic validation. In the current work, we focused on integrating clinical data, inflammation-driven animal models, and gain-of-function analyses to define the role of SLPI in cholangiocarcinoma progression. The consistency of SLPI-associated phenotypes observed across human transcriptomic datasets, hamster and mouse models, and multiple in vitro assays supports the biological relevance of SLPI despite the absence of loss-of-function experiments.  As noted in the revised Discussion, the lack of SLPI loss-of-function studies represents a limitation of this work. We have explicitly highlighted SLPI knockdown, neutralization, and rescue experiments as important directions for future investigation to further strengthen causal inference. |
| 3 | Line 103: Provide details of the total number of mice and how they were grouped and allotted for each analysis. | We thank the reviewer for this comment and agree that clearer reporting of animal numbers and group allocation improves transparency.  In the revised manuscript, we have clarified the total number of animals used, experimental grouping, and allocation for each analysis. In the inflammation-induced hamster model, animals were divided into four time-point groups (0, 1, 3, and 6 months), with three animals per time point used for immunohistochemical analyses of SLPI expression. In the mouse xenograft model, male nude mice were randomly assigned to two groups (Mock and SLPI-overexpressing cells), with nine mice per group. All animals were monitored according to predefined humane endpoint criteria.  These details have now been explicitly stated in the Methods section. |
| 4 | Line 132, 139,144: For cell culture experiments, although initially three cell lines have been mentioned, it is unclear which cells were used for each analysis and there are no details on the different groups or how the treatment was done. Please include details of this in the methodology section. | We thank the reviewer for this important comment regarding clarity of cell line usage and experimental grouping.  In the revised manuscript, we have clarified the specific roles of each cell line and explicitly stated which cells were used for each experimental assay. MMNK-1 cells were used for inflammation modeling and SLPI overexpression experiments, including functional gain-of-function analyses. KKU-213A and KKU-213AL5 cholangiocarcinoma cell lines were included as CCA models with endogenous SLPI expression for comparative analyses. EA.hy926 endothelial cells were used exclusively for angiogenesis-related assays.  In addition, experimental grouping and treatment conditions (e.g., Mock vs. SLPI-overexpressing cells, IL-6 stimulation where applicable) are now clearly described at the beginning of each relevant assay subsection in the Methods. These revisions were made to improve transparency and reproducibility without altering the experimental design or conclusions. |
| 5 | Line 139, 144, 168, 174: Wherever cell count is mentioned express them as cells /ml or cell /cm2 or cell/well. | We thank the reviewer for this comment and agree that consistent reporting of cell seeding density improves clarity and reproducibility.  In the revised manuscript, we have standardized the reporting of cell numbers throughout the Methods section. Cell seeding densities are now consistently expressed as cells per well (cells/well), cells per milliliter (cells/mL), or cells per square centimeter (cells/cm²), as appropriate for each experimental format. These revisions were made to improve methodological clarity without altering the experimental design. |
| 6 | Line 145: How were the cytosolic proteins obtained for ELISA assay. | We thank the reviewer for this comment and agree that clarification of cytosolic protein preparation is necessary.  In the revised manuscript, we have explicitly described the procedure used to obtain cytosolic proteins for ELISA analysis. Briefly, cells were harvested, washed with cold PBS, and lysed using RIPA buffer supplemented with protease inhibitors. Cell lysates were clarified by centrifugation to remove cellular debris, and the supernatants were collected as cytosolic protein fractions. Protein concentrations were determined prior to ELISA analysis to ensure equal loading.  This clarification has now been added to the Methods section. (pages 7, line 148-157) |
| 7 | Line 105: The number of mice, animal grouping with respective treatment needs to be mentioned. | We thank the reviewer for this comment and agree that clear reporting of animal numbers, grouping, and treatment allocation is essential.  In the revised manuscript, we have explicitly clarified the number of animals, experimental grouping, and treatment conditions in both animal models used in this study. For the inflammation-induced hamster model, animals were grouped according to predefined time points (0, 1, 3, and 6 months), with three animals per time point used for immunohistochemical analysis of SLPI expression. For the mouse xenograft model, male nude mice were randomly assigned to two groups (Mock and SLPI-overexpressing cells), with nine mice per group. The respective treatments and humane endpoint criteria are now clearly stated in the Methods section. (pages 5, line 106; pages 10, line 212-224) |
| 8 | Line 164: Was 5 mg/ml, the final MTT concentration in wells? | We thank the reviewer for this comment and agree that clarification of the MTT concentration is necessary.  In the revised manuscript, we have clarified that 5 mg/mL refers to the final concentration of MTT reagent in the wells during the assay. This information has now been explicitly stated in the Methods section to avoid ambiguity. (pages 7-8, line 161-162) |
| 9 | Figure 1a-b: Include MW markers | We thank the reviewer for this comment and agree that inclusion of molecular weight (MW) markers improves clarity and data interpretation.  In the revised manuscript, molecular weight markers have now been added to the Western blot images in Figure 2a–b. The corresponding MW values for SLPI, p65, p-p65 and loading control proteins are also indicated in the revised figure to facilitate interpretation. These changes improve figure clarity without altering the underlying data. |
| 10 | Figure 2c-f: It may be more appropriate to represent the Y axis of western blot graphs in this format relative expression (fold change) of pNF-κB | We thank the reviewer for this helpful suggestion and agree that labeling the Y-axis as relative expression (fold change) improves clarity and interpretability.  In the revised manuscript, we have updated the Y-axis labels of the Western blot quantification graphs in Figure 2c–f to indicate **relative expression (fold change**) of phosphorylated NF-κB, normalized to total NF-κB and the loading control. The figure legends have also been revised accordingly to clearly describe the normalization and quantification approach. (Figure 2c-f and figure legend) |
| 11 | Fig 5f: Provide the molecular weight of zymogram bands  in studies investigating its function. | We thank the reviewer for this helpful comment. We agree that indicating the molecular weights of gelatin zymogram bands improves clarity and interpretation.  In the revised manuscript, we have added molecular weight (MW) annotations to the gelatin zymography image in Figure 5f to indicate the positions of pro-MMP-9 (~92 kDa), active MMP-9 (~82 kDa), pro-MMP-2 (~72 kDa), and active MMP-2 (~62 kDa). The Figure 5 legend has also been revised accordingly. These additions improve figure clarity without altering the underlying data. (Figure 5f) |
| 12 | Line 257: If IL6 treatment can upregulate SLPI expression and activate pathway, why was it not preferred SLPI over overexpression | We thank the reviewer for this insightful comment. We agree that IL-6 is a physiologically relevant inflammatory stimulus in cholangiocarcinoma and therefore included IL-6 treatment experiments in this study to model inflammation-driven SLPI induction.  However, SLPI overexpression was employed as a complementary approach to specifically delineate the functional contribution of SLPI independent of upstream inflammatory signaling. IL-6 activates multiple downstream pathways, including NF-κB and STAT3, and regulates numerous target genes beyond SLPI. Thus, while IL-6 treatment is suitable for modeling inflammatory induction, it does not allow isolation of SLPI-specific effects on tumorigenic phenotypes.  By combining IL-6 stimulation with SLPI gain-of-function experiments, our study was designed to distinguish between (i) inflammation-induced SLPI upregulation and (ii) the direct tumor-promoting functions of SLPI itself. The concordant phenotypes observed under both conditions support the conclusion that SLPI is not merely a bystander of inflammation but a functional mediator of cholangiocarcinoma progression.  This rationale has now been clarified in the revised Discussion section. |
